# Improving Access to and Quality of Addiction Training Opportunities in the West Midlands: A Quality Improvement Project

**DOI:** 10.1192/bjo.2026.11289

**Published:** 2026-06-30

**Authors:** Oluwafemi Popoola, Derrett Watts

**Affiliations:** 1 North Staffordshire Combined Healthcare NHS Trust, Stoke-on-Trent, United Kingdom; 2 Birmingham and Solihull Mental Health Foundation Trust, Birmingham, United Kingdom

## Abstract

**Aims:**

In the West Midlands, limited addiction placements and a shortage of suitably endorsed assessors have made it difficult for core psychiatry trainees to meet revised RoyalCollege of Psychiatrists (RCPsych) curriculum requirements, including completion of two addiction-specific workplace-based assessments (WPBAs). Without targeted intervention, trainees risk failing to demonstrate required competencies, with implications for future specialist workforce capacity as there continue to be fewer addiction psychiatrists working in substance misuse services.

Aims were to improve access to, and quality of, addiction psychiatry training opportunities for core psychiatry trainees in the West Midlands, with a particular focus on enabling completion of curriculum-mandated addiction workplace-based assessments (WPBAs).

**Methods:**

Baseline data were obtained from a cross-sectional review of ARCP outcomes in December 2024 which showed trainees were struggling to meet their addiction workplace-based assessment (WPBA) requirements. We focused on four intervention methods: (1) assessor training in completing addiction workplace-based assessments (WPBAs), (2) development of a regional assessor network and addiction training plan for each Trust in the region, (3) experience and special interest days within addiction services with opportunities to complete assessments, and (4) expansion of addiction teaching within the MRCPsych programme.

Outcome and process measures included proportion of trainees meeting addiction WPBAs requirements, number of trained assessors, availability of more learning opportunities, and trainee feedback.

**Results:**

At baseline, none of 13 core trainees in the baseline data had completed the required two addiction WPBAs, although 11 had completed one. Assessor training sessions were organised which generated positive feedback and volunteers leading to the development of anaddiction training plan for each Trust with directory of assessors. Experience days enabled trainees to complete assessment of clinical expertise (ACEs) and case-based discussions (CBDs) with appropriate supervision and were associated with improved confidence and engagement with addiction psychiatry. By the June 2025 ARCP, all CT3 trainees had completed the required addiction WPBAs.

**Conclusion:**

This project shows that practical, system-level changes can make a meaningful difference to trainees’ access to addiction training and their ability to meet curriculum requirements. In particular, developing assessor capability, prioritising experiential learning, and working collaboratively at a regional level were central to progress. Ongoing senior leadership is needed to sustain this momentum so that the changes can be embedded into routine practice, and to ensure equitable access across regions.

